# The self-reinforcing cycle under dual constraints—a study of the behavioral logic of urban empty-nest older adults substituting self-medication for formal medical treatment

**DOI:** 10.3389/fpubh.2026.1701771

**Published:** 2026-03-05

**Authors:** Ji Wu, Yuanyuan Liu, Siwen Li

**Affiliations:** School of Humanities and Social Sciences, Harbin Engineering University, Harbin, China

**Keywords:** disease response, grounded theory, health sociology, substituting self-medication for formal medical treatment, urban empty-nest older adult

## Abstract

**Introduction:**

Against the stark backdrop of China’s rapidly aging population and the high prevalence of chronic diseases among the older adults, urban empty-nesters face a dual challenge stemming from inadequate intergenerational support and heightened health vulnerability. A deeper understanding of their illness coping behavior is therefore critically needed.

**Methods:**

This study employs grounded theory methodology, conducting in-depth interviews with 25 empty-nest older adults in H City. A total of 290,000 words of interview texts were collected to generate a theoretical model, systematically revealing the individual cognitive framework and dynamic mechanisms of health behaviors within this group.

**Results:**

The study reveals a “dual constraints–trigger mechanism–self-reinforcing cycle” logic underpinning the health coping strategies of empty-nest elders: (1) At the precondition level, insufficient intergenerational support and self-perceived burden constitute structural dual constraints; (2) The trigger mechanism is activated by illness experience and self-diagnosed health anxiety, which interact and amplify each other through lay explanatory models of illness, thereby directly driving behavioral decisions; (3) The behavioral pathway follows a self-reinforcing sequence of “self-medication → consulting community networks for medication advice → seeking medical treatment,” further exacerbated by structural contradictions within the healthcare system; (4) Outcomes form a closed loop, in which these health management practices not only conclude the current coping cycle but also often initiate new health issues, restarting the entire cycle.

**Discussion:**

The findings indicate that the behavior of “substituting self-medication for formal medical treatment” essentially functions as a survival strategy among the older adults under structural constraints. This strategy allows them to maintain autonomy through self-reliant narratives and achieve social adaptation via peer group-based mutual support. The study offers a theoretical pathway that integrates structural reflection with subjective care, contributing meaningfully to the development of healthy aging policies.

## 1 Introduction

Population aging represents a profound social challenge, not only in China but globally. By the end of 2024, the number of individuals aged 60 and above in China will exceed 280 million, constituting 22% of the total population, and this trend is expected to continue. Simultaneously, health issues among the older adults have become increasingly pronounced. According to the data from the National Health Commission of China, over 180 million older adults suffer from chronic diseases, with 75% affected by at least one chronic condition, underscoring the pressing need to advance healthy aging strategies (1).

In this context, the health challenges faced by empty-nest older adults, who make up a significant portion of the older adult population, are particularly severe. Studies have shown that empty-nest older adults experience notably poorer health compared to their non-empty-nest peers (2). Devoid of emotional support and daily healthcare, this demographic attribute faces higher rates of chronic diseases and comorbidities, resulting in greater health risks and increased medical needs (3, 4). The illness coping behavior of empty-nesters, along with its underlying mechanisms, plays a crucial role in determining their health outcomes.

At present, the number of empty-nesters in China has exceeded half of the total older adult population. The proportion of older adult empty-nest families in large and medium-sized cities in China has reached 70%. With the continuous advancement of urbanization, the proportion of urban empty-nest older adults is expected to rise further (5). Therefore, it is of great significance to explore the health status of urban empty-nesters in China, systematically analyze their disease coping styles, and reveal their behavioral logic and key influencing factors to improve their disease coping behaviors, improve their health level, optimize the supply of health services, and improve the medical care system for the older adults. It will also provide a solid basis for promoting the formulation of policies related to healthy aging and active aging^1^.

## 2 Literature review

The concept of the “empty-nest” originates from family life cycle theory (6). Research indicates that empty-nest older adults face significant psychological and physiological health challenges. Their mental health and self-reported health status are considerably worse than those of their non-empty-nest counterparts (2), and they exhibit higher rates of anxiety and illness (7). Furthermore, due to physiological decline and limited social support, empty-nest older adults are more vulnerable to various chronic non-communicable diseases (8). It is also crucial to consider the potential spillover effects of their psychological health on their physical health (9).

Illness coping behavior refers to an individual’s response to illness, which primarily involves self-medication and seeking medical treatment. Self-medication includes practices such as self-prescribing, self-care, and self-management (10). The prevalence of self-medication is increasing among residents in China, particularly in urban areas (11). Among the older adults, self-medication is common as a means of alleviating economic burdens (12). Still, the lack of medication knowledge often results in frequent and varied drug use (13), posing potential health risks (14). In terms of self-care and self-management, older adults often rely on regular routines and physical exercise (15). In recent years, digital health management has emerged (16) and may enhance health awareness (17). Still, it is accompanied by problems such as information interference, which hinders their efficient access to and understanding of information and may delay medical treatment for empty-nesters with declining learning ability and children leaving home (18).

It is worth noting that self-medication is often associated with delays in seeking medical treatment (19). In China, delayed medical visits among chronic disease patients are widespread, with incidence rates ranging from 26 to 90%. Such delays can result in missed diagnoses, reduced treatment efficacy, and a decline in quality of life (19, 20). The mindset among the older adults of putting off minor illnesses and bearing serious ones (21), which increases the likelihood of untreated conditions.

Numerous factors influence the medical-seeking behavior of empty-nest older adults. The intergenerational support is a key factor, as economic and caregiving assistance can significantly increase the likelihood of older adults seeking treatment (22). Proximity to healthcare institutions also plays a significant role; easier access to medical services increases the likelihood of seeking medical treatment (23). Significant regional disparities exist, leaving rural empty-nest seniors grappling with both elevated disease prevalence and inferior access to healthcare (24). Additionally, an individual’s health status and the nature of their diseases (such as chronic conditions) also influence their willingness to seek medical treatment (20). Broader issues within the Medical Social Ecology, such as the professional ethics alienation and the strained doctor-patient relationship, further complicate the illness coping behavior of older adults (25).

In terms of theoretical model construction, the Health Belief Model offers a theoretical lens for understanding how older adults construct subjective meanings as they perceive disease severity, assess personal susceptibility, and evaluate the benefits and barriers of taking action (26). By emphasizing the central role of cognitive factors in initiating health behaviors, this model provides a micro-level psychological framework for analyzing the subjective motivations underlying older adults’ health-related decisions. Anderson’s Health Service Utilization Model systematically categorizes factors influencing healthcare-seeking behavior into three types: propensity characteristics, enabling resources, and demand factors. Propensity characteristics encompass demographic and social attributes such as age, gender, and cultural background; enabling resources include external support conditions like economic income, health insurance coverage, and healthcare accessibility; Demand factors refer to an individual’s health status and subjective need for medical care, emphasizing the shaping role of social environments and resource allocation on health behaviors. This framework provides a reference for comprehensively examining the structural constraints on healthcare decision-making among empty-nest older adult individuals in this study (27).

Although existing research has focused on the health problems of urban empty-nesters, it is largely limited to quantitative analysis of specific behaviors such as self-medication and medical delay, and their external determinants. It lacks an in-depth analysis of the dynamic response process of empty-nesters following disease onset from a holistic perspective and pays less attention to the role of older adults’ own cognition and meaning construction. Existing theoretical models predominantly focus on the influence of individual cognition on health behaviors or static analyses of multiple factors, failing to adequately explain how older adults within the specific family structure of empty-nest households and the Chinese social context dynamically integrate, interpret, and respond to structural health threats within their subjective cognitive frameworks. Building on this context, this study employs grounded theory methodology to construct a coping behavior model grounded in the lived experiences of urban empty-nest older adults in China, with a focus on the “logic of illness coping behaviors.” Rooted in the local realities of this population, the study systematically examines the dynamic formation process, cyclical reinforcement mechanisms, and the exercise of agency in older adults’ illness coping within specific family structures and cultural contexts. This approach represents a theoretical advancement by shifting from universal frameworks to contextually adaptive models and moving beyond factor analysis toward mechanism-based interpretations. Ultimately, it provides a theoretical foundation for developing health support policies that are more context-sensitive and person-centered.

## 3 Research methods and data collection

### 3.1 Research methods

This study adopts a grounded theory research methodology. Semi-structured in-depth interviews were conducted to collect primary data, and the analysis followed the strict procedures outlined in grounded theory: the transcribed texts were initially open-coded to identify key concepts; axial coding was employed to explore relationships between these concepts and form core categories; finally, selective coding was applied to integrate the categories and construct a comprehensive theoretical model. This method aims to systematically uncover the underlying mechanisms driving illness coping behaviors among urban empty-nest older adults, thereby addressing the gaps in existing theoretical research.

### 3.2 Data collection

The research focused on urban empty-nest older adults in District L of City H. A community-based random sampling method was employed, yielding 25 eligible participants for in-depth semi-structured interviews. Each interview lasted between 40 and 60 min. As shown in Table 1, respondents’ self-rated health status predominantly clustered around “average” and “poor,” reflecting the typical self-assessment of health among urban seniors and providing robust empirical support for the extraction of core categories. The diversity in marital status and number of children, meanwhile, facilitates analysis of commonalities in disease coping strategies among seniors across different family support contexts, thereby enhancing the model’s applicability to China’s community-dwelling older adult population. This study strictly follows the ethical norms of scientific research. Before the interview, all participants were informed of the purpose, content, and data use of the study, and their informed consent was obtained. All interview data were anonymously coded to protect the privacy of the respondents. The research program has been approved by the Ethics Committee of the School of Humanities and Social Sciences of Harbin Engineering University. Following the verbatim transcription of the recordings, approximately 290,000 words of textual data were obtained. In accordance with grounded theory data analysis principles, 20 interviews were randomly selected for comprehensive three-level coding (open coding, axial coding, and selective coding) and theoretical model construction. The remaining five interviews were set aside for subsequent testing of theoretical saturation.

**Table 1:** Basic information of interview subjects.

Serial number	Gender	Age	Marital status	Number of children	Self-assessment of health
R1	Female	90	Widowed	3	Poor
R2	Female	74	Widowed	1	Average
R3	Male	71	Married	1	Fairly poor
R4	Female	68	Married	1	Average
R5	Female	71	Married	1	Poor
R6	Male	67	Married	1	Average
R7	Female	66	Married	2	Average
R8	Male	66	Married	1	Average
R9	Male	70	Married	1	Average
R10	Female	65	Remarriage	1	Poor
R11	Female	72	Divorced	1	Fairly good
R12	Male	66	Married	1	Average
R13	Female	70	Married	2	Average
R14	Male	78	Divorced	2	Fairly poor
R15	Female	64	Single	0	Average
R16	Female	70	Married	1	Average
R17	Female	74	Widowed	1	Average
R18	Male	68	Married	1	Average
R19	Male	76	Married	1	Poor
R20	Female	65	Married	2	Fairly good
R21	Female	71	Widowed	1	Average
R22	Male	76	Widowed	1	Poor
R23	Female	69	Divorced	2	Average
R24	Female	77	Married	1	Average
R25	Male	64	Married	1	Average

### 3.3 Data coding and model construction

#### 3.3.1 Open coding

Open coding refers to the process of breaking down, examining, and comparing the raw data to generate concepts and categorize them (28). In this study, the interview transcripts were manually coded, yielding 224 initial labels through detailed reading and analysis. These labels were subsequently merged into 46 initial concepts and 17 subcategories (as shown in Table 2).

Taking the formation process of the initial concept “Hospital treatment is not effective” as an example. During sentence-by-sentence coding, researchers first identified 11 original statements, such as R2 mentioning “The hospital makes you do this and that, but the tests still show the same conditions I had before,” and R7 stating “I went to the hospital for a headache check, but they found nothing.” These were labeled as “ineffective examinations.” Simultaneously, eight other statements—including R8’s “Not worth the registration fee; asking questions is useless” and R10’s “Better to ask yourself than the doctor”—were labeled as “distrust of doctors.” Through in-depth analysis of the interview data, researchers discovered that “distrust of doctors” often stemmed from prior experiences of “ineffective examinations,” revealing a causal relationship between the two. Consequently, these two labels were synthesized into the initial concept “Hospital treatment is not effective”.

**Table 2:** Open coding and categorization.

Subcategory	Initial concept	Original representative statements in the interview text
B1 Lack of emotional support	A1 Not coming often	R2 Children are busy at work, how can he be with you every day?
	A2 children are out of town	R7 Children are working in Sanya.
B2 Lack of material support	A3 Low savings	R16 We do not make a lot, so let us live within our means and just focus on taking good care of ourselves.
	A4 Adding financial burden on children	R23 Getting sick always costs an arm and a leg. Our kids do not make much to begin with, so this is just piling more on their plate.
B3 Self-supporting life	A5 Children’s work pressure	R2 Now you cannot say that your children are not filial, the pressure of work is too great.
	A6 Do not add to your children’s burden	R18 Now that I’m old, I can take care of myself, and I do not want to cause trouble for my children.
B4 Multiple health problems	A7 Suffering from chronic or other diseases	R19 Besides diabetes, there is high blood pressure.
	A8 Suffering from multiple diseases	R25 High blood pressure, neurasthenia, and a bit of depression, insomnia.
B5 Afflicted with ailments and pains	A9 Disruption to normal life	R15 Vertigo the sky is spinning and I cannot function normally.
	A10 Sickness is hard to bear	R7 Trigeminal neuralgia hurts so much
	A11 Long duration of illness	R24 Remember when I was in my forties I was confused and took medication without knowing what was wrong.
	A12 Life without hope	R10 When you are in pain, you do not want to live, it’s all meaningless.
B6 Autonomous illness perception	A13 Autonomous attributions	R22 Working night shifts back in the day totally messed up my sleep.
	A14 Negative associations	R5 When you cannot sleep well problems all come.
B7 Illness anxiety	A15 Avoidance of medical treatment	R12 Sometimes, getting tests done just lands you with a huge bill and more stress. You’re better off not knowing!
	A16 must take medication	R14 I have no choice but to take the medicine; otherwise, it’s just gonna get worse.
	A17 there is no hope for a cure	R1 Nothing more we can do (about these health problems). Just getting old, I guess.
B8 Medication stockpiling	A18 Drug reserve	R3 It’s all meds. When you get to a certain age, you gotta have some pills on hand just in case. Do not wanna be caught off guard.
	A19 Reason for stockpiling	R14 These days I’m figuring I should save up, for when I really cannot take care of myself anymore.
B9 Self-medication practices	A20 Undiagnosed medication	R9 Never really got a diagnosis. Just taking the pills, whatever.
	A21 Self-dispensing medication	R4: I take AXX and OXX, Sometimes I have some of that GXX, And if my blood pressure goes up, I’ll toss in some GXX.
	A22 Self-adjustment of medication frequency	R16 Three times a day, I usually take two times a day.
	A23 Alternate medication	R10 I alternate between these three meds so I do not become dependent
	A24 Not taking the prescribed dosage	R16 Do not even bother reading the instructions. If your cold gets really bad, just take more.
	A25Multiple medications	R13 Besides the OXX, I’m also on GXX for my angina, plus meds for my blood pressure.
	A26 drug accessibility medication	R5 Zopiclone is hard to get, but EXX and AXX can be found easily, so I usually buy EXX and AXX.
B10 Peer-based medication inquiry	A27 Peers recommend drugs to each other	R18 (Peers) Take care of each other, I take that drug, you take it too.
	A28 Peers recommend alternative medical treatment information	R14 Actually, trying that moxibustion chair was my sister’s idea. You know, most people aren’t really keen on going to the hospital; they’d rather go for a moxa session.
B11 Community-based medication inquiry	A29 Pharmacy asks for medicine	R7 I’ll just go down to the pharmacy and buy it.
	A30 Prescription at the community clinic	R21 Basically at the community clinic.
B12 Online medication inquiry	A31 Short video to be informed about the medicine	R15 I saw something about a medication for that on TikTok. I’ll check it out at the drugstore sometime and maybe give it a shot.
	A32 Webcasting to buy medicine	R17 I watched a platform live stream that sold medicine, so I bought a bottle and came back to take it.
B13 Reluctance to seek medical treatment	A33 Seek medical treatment for major illnesses	R2 A big problem, you cannot survive it, that’s why you have to go to the hospital.
	A34 Forced to seek medical treatment	R11 Unless it hurts a lot (cannot stand it), you go to the hospital.
	A35 Medical insurance does not work	R1 My insurance pays for the hospitalization, but the meds are all out-of-pocket, I think.
	A36 Hospital treatment is not effective	R7 When I go to the hospital, I say my brain hurts, but I cannot find out anything when I go to the hospital.
	A37 It’s hard to see a doctor	R16 I went on the first day and was told to go the next day, I went at 7:00 the next day and was not seen until 10:00.
B14 Medical treatment demand	A38 Wanted to go for medical treatment	R13 I’ve had this cough for years, and my son told me to find a big hospital to look at it.
	A39 Long-time illness not checked	R5 The neurasthenia problem started when I was young, I have not been to the hospital.
B15 Health monitoring	A40 Regular medical checkups	R21 After we both retired, we went for medical checkups almost every day as partners.
	A41 Measurement of blood pressure/blood sugar	R2 Measure it in the morning and the evening.
B16 Daily healthcare	A42 Have a healthy diet	R19 Bananas are full of sugar, so you cannot eat too many of them.
	A43 Take health supplements	R3 I take vitamin D and this calcium supplement.
	A44 Physical Medical treatment in Chinese medicine	R16 I went to a Chinese medicine center to adjust the blood supply to my brain and heart, and 3 months later I had my kidneys adjusted.
B17 Learning about health	A45 Watching healthcare programs	R12 I usually watch CCTV healthcare programs, more or less learn a little.
	A46 Listening to health lectures	R13 In the community I go over there, it happens to be a sleep lecture I go to see.

#### 3.3.2 Axial coding

Axial coding seeks to deepen the categories identified through open coding and to uncover the inherent logical relationships between them, thereby illustrating the interconnectedness of various elements within the original data (29). In this study, a systematic analysis of the 17 subcategories was conducted to examine their conceptual and behavioral relationships. These subcategories were clustered and integrated to clarify further and reinforce the connections between categories. Ultimately, eight core categories (C1–C8) were derived and grouped into four primary dimensions (as shown in Table 3).

**Table 3:** Axial coding.

Dimension	Core categories	Subcategories
Preconditions	C1 Insufficient intergenerational support	B1 Lack of emotional support
	C2 Self-perceived burden	B2 Lack of material support
	C3 Independent living	B3 Self-supporting life
Trigger mechanism	C4Subjective illness experience	B4 Multiple health problems
		B5 Afflicted with ailments and pains
	C5 Self-diagnosed health anxiety	B6 Autonomous illness perception
		B7 Illness anxiety
Behavioral pathway	C6 Self-medication	B8 Medication stockpiling
		B9 Self-medication practices
	C7 Consulting community networks for medication advice	B10 Peer-based-medication Inquiry
		B11Community-based medication inquiry
		B12 Online medication inquiry
	C8 Seeking medical treatment	B13 Reluctance to seek medical treatment
		B14 Medical treatment demand
Result feedback	C9 Daily health management	B15 Health monitoring
		B16 Daily healthcare
		B17 Learning about health

#### 3.3.3 Selective coding and model construction

Selective coding focuses on exploring the internal logical relationships between core categories, integrating the interconnected categories to extract a central category that synthesizes all data. Based on the analysis of relationships between the core categories, this study constructs the core storyline of the illness coping behavior logic among urban empty-nest older adults.

The illness coping behavior of urban empty-nest older adults begins with “Insufficient Intergenerational Support” and “Sense of Self-Burden,” which serve as preconditions. These dual constraints establish the underlying logic for their behavioral choices: The insufficient intergenerational support directly limits the older adults’ access to external resources, while the internalized “Sense of Self-Burden” further diminishes their willingness to seek external help. Under these preconditions, “illness experience” and “self-diagnosed health anxiety” act as the triggering mechanism, mutually reinforcing each other. That is, physical discomfort triggers or intensifies health concerns, and this anxiety leads individuals to interpret and evaluate their symptoms more negatively. The combined effect of these factors directly drives specific behavioral pathways for disease management. Specifically, their coping behavior follows a self-reinforcing cycle of “Self-medication → Consulting community networks for medication advice → Seeking medical treatment.” This pathway is not strictly linear, as individuals may cycle through these stages depending on the situation. Ultimately, the feedback from these behavioral choices influences daily health management practices. These outcomes possess dual attributes: they signify the end of the current coping cycle, while also initiating new health problems that restart the cycle.

**Figure 1:**
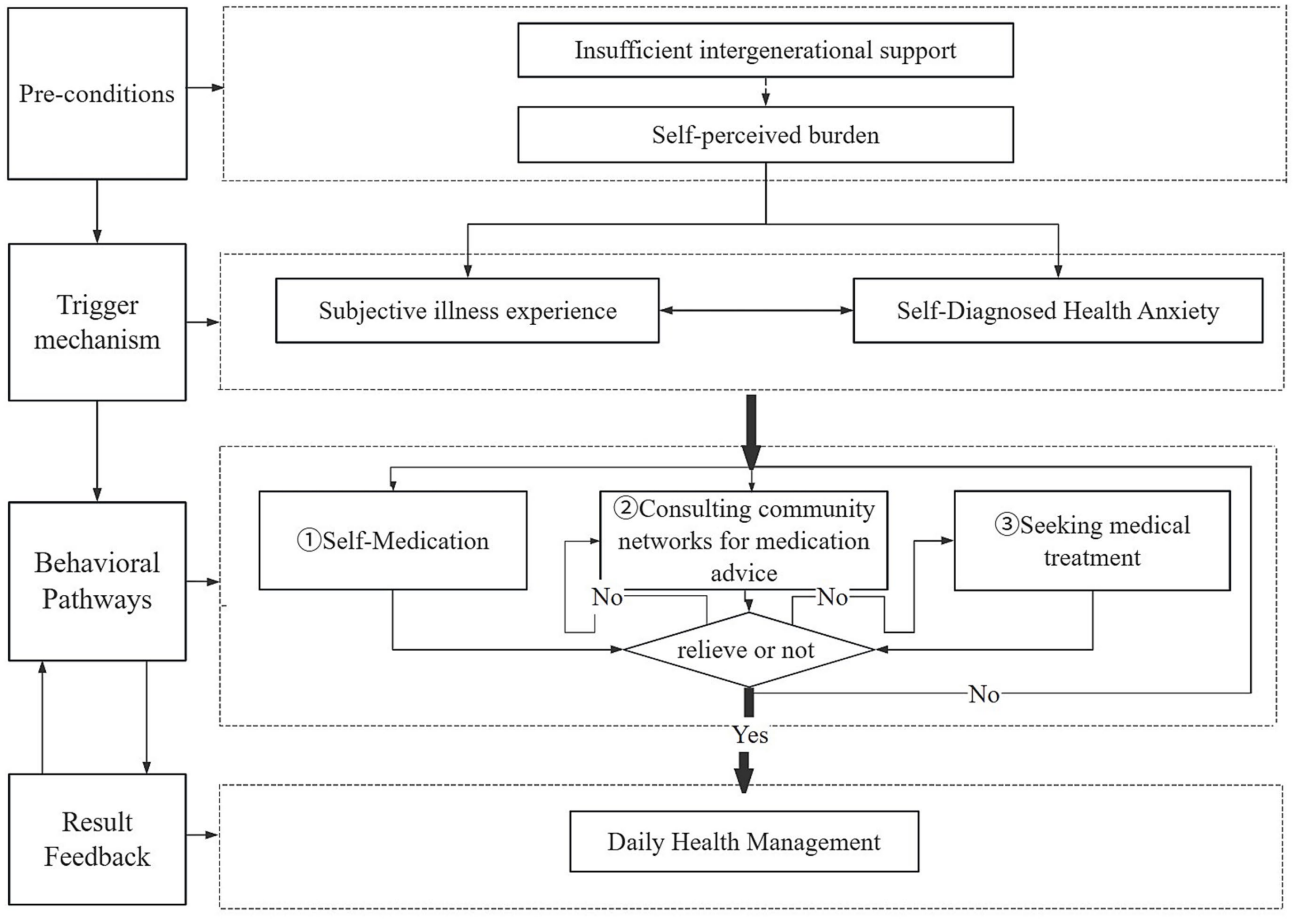
Generation mechanism of illness coping behavior in urban empty-nest older adults.

This study establishes the “generation of illness coping behavior in urban empty-nest older adults” as the core category. This category effectively integrates all the concepts, subcategories, and core categories that emerged from the original data, successfully incorporating the dynamic storyline above and ultimately forming a theoretical framework model to reveal the intrinsic mechanisms behind the illness coping behavior of urban empty-nest older adults (as shown in Figure 1).

#### 3.3.4 Saturation test

To test the saturation of the conceptual model, this study employed repeated coding on the five previously prepared text materials. No new concepts or hierarchical relationships were identified, indicating that the conceptual framework and mechanism model have essentially achieved theoretical saturation. Throughout the study, an external review panel comprising three senior health sociology researchers who did not participate in the coding process regularly attended coding discussions and category refinement meetings. This ensured the accuracy of category refinement and the explanatory power of the model.

## 4 Interpretation of the illness coping behavior generation model of urban empty-nest older adults

### 4.1 Pre conditions—dual constraints of insufficient intergenerational support and sense of self-burden

The illness coping behavior of empty-nest older adults does not emerge in isolation; it is grounded in profound and interrelated preconditions. These preconditions form the foundational logic behind their behavioral choices and establish the basic framework for subsequent illness coping behaviors. Based on grounded theory analysis, this study reveals that the illness coping behavior of urban empty-nest older adults is strongly influenced by the dual preconditions of “Insufficient intergenerational support” and “Sense of Self-Burden.” Both as preconditions are the root causes of the older adult’s behavior of seeking medical treatment and medicine, and are deeply influenced by the deep-rooted traditional concept of “raising children for old age” in China (30, 31).

#### 4.1.1 Insufficient intergenerational support—dual lack of material and emotional support

Intergenerational support refers to the resource exchange between children and older adult parents within a family, primarily flowing from children to parents (32). It encompasses both the economic and emotional support (33). In this study, the insufficient intergenerational support is defined as the condition where empty-nest older adult experience both inadequate material and emotional support during their illness coping process. Some interviewees actively minimize their economic dependence on their children due to limited financial resources or a reluctance to impose a burden on them, as exemplified by the respondent R16: “We do not make a lot, so let us live within our means and just focus on taking good care of ourselves.” This economic pressure prompts the older adults to favor low-cost treatment options when coping with disease. Additionally, the interview data reveal that children typically fail to engage in meaningful emotional exchanges with their older adult parents. The absence of emotional support impedes the older adults from completing necessary illness narratives through channels like confiding in their children or getting companionship, thereby hindering their ability to effectively alleviate health anxiety or express health concerns. The insufficient intergenerational support not only limits the older adult’s access to external resources but also places them in a state of both social and psychological isolation during their illness coping.

#### 4.1.2 Self-perceived burden—internalized psychological constraints

Self-perceived burden refers to the psychological states of guilt, distress, responsibility, and diminished self-worth that arise when an individual feels their illness and care needs impose a burden on others (34). Specifically, this sense of burden encompasses three interrelated aspects: older adults perceive their physical care needs as imposing on others, experience guilt stemming from dependence and diminished self-worth, and worry that their medical and care expenses will place financial strain on their families (35). Research shows that some older adults, concerned about adding to their children’s financial burdens, opt to endure health problems in silence. As the respondent R23 stated, “Getting sick always costs an arm and a leg. Our kids do not make much to begin with, so this is just piling more on their plate.” This internalized psychological pressure prevents older adults from actively seeking help from their children, even when faced with significant health challenges. Many older adults view “I do not want to be a burden on my kids” as a form of support, believing that by shouldering their own health struggles, they are alleviating the strain on their family.

#### 4.1.3 Interactive reinforcement mechanism of dual constraints

The insufficient intergenerational support and Self-perceived burden do not exist independently; rather, they form a mutually reinforcing dual constraint. The insufficient intergenerational support places the older adults in a state of both material and emotional deprivation. At the same time, neglect caused by them intensifies the sense of self-burden, manifesting as a strong sense of guilt. This “guilt”—the feeling of being a burden on their children—significantly inhibits their willingness to seek help, objectively worsening the intergenerational support, thereby creating a dilemma of “no resources available” and “unwilling to use available resources.” Under this dual constraint, the older adults’ illness coping behavior tends to reflect a passive response inclination. This dual-constraint mechanism not only reveals the structural dilemma underlying the behavior of empty-nest older adults but also provides a theoretical foundation for exploring their specific behavioral pathways.

### 4.2 Trigger mechanism—interaction between illness experience and health anxiety

The trigger mechanism of illness coping behavior is the core link between the preconditions and subsequent behavioral pathways. This study finds that, under the influence of these dual preconditions, the illness coping behavior of urban empty-nest older adults is primarily driven by the interaction between “illness experience” and “self-diagnosed health anxiety” (hereafter referred to as “health anxiety”), which directly influences their behavioral choices.

#### 4.2.1 Illness experience—embodied perception of physical discomfort

The illness experience encompasses an individual’s physical symptoms caused by illness, subjective feelings, meaning interpretation, and their interaction with social and cultural contexts (36, 37). It extends beyond a mere physiological response, highlighting the perception and construction of bodily changes and their meanings within a social and cultural framework (38). The interview data reveal that illness brings the “disappearing” body “Presencing” (39), amplifying the embodied experience of pain. This not only disrupts the older adults’ daily life (e.g., limiting activities and reducing functional capacity) but also fosters pessimistic expectations about the disease’s long-term consequences. As the respondent R1 stated: “Nothing more we can do (about these health problems). Just getting old, I guess.” This experience lays the physiological and psychological foundation for triggering illness coping behavior.

#### 4.2.2 Self-diagnosed health anxiety—cognitive bias and emotional negative cycle

The research data reveal that the empty-nest older adults tend to interpret their symptoms through self-attribution, as exemplified by the respondent R22’s statement: “Working night shifts back in the day totally messed up my sleep,” or through negative associations, such as the respondent R5’s statement: “Not getting enough sleep is bad for my heart and head.” These non-scientific interpretations of symptoms contribute to the development and intensification of health anxiety. Health anxiety manifests as excessive sensitivity to illness, dependence on medication (e.g., R14: “I have no choice but to take the medicine; otherwise, it’s just gonna get worse.”), avoidance of medical treatment (e.g., R12: “Sometimes, getting tests done just lands you with a huge bill and more stress. You’re better off not knowing!”), and even a pessimistic belief that the condition is incurable (e.g., R1: “It’s hopeless. That’s just the way it is at my age.”). The interaction between anxiety and physical symptoms creates a negative feedback loop, amplifying the perception of even minor discomforts.

#### 4.2.3 Interactive driving mechanism of behavior

Disease explanation models can be divided into two categories: professional (biomedical) and public (folk explanations and patients’ subjective experiences) frameworks (40). This study finds that illness experience and health anxiety form a dynamic, mutually reinforcing relationship through the lay illness explanatory models. Specifically, the illness experience, when interpreted through a non-expert and non-professional lens, exacerbates the health anxiety. In turn, the health anxiety magnifies the over-interpretation of bodily symptoms and leads to catastrophic evaluations, further intensifying the perception of discomfort. This reciprocal, the cyclical interaction—using the lay illness explanatory model as a cognitive bridge—not only transmits the negative impact of physiological discomfort on psychological states but also shapes the individual’s hypersensitive interpretation of bodily experiences, ultimately becoming the direct driving force behind specific coping behaviors.

### 4.3 Behavioral pathway—self-reinforcing cycle from self-medication to seeking medical treatment

Under the direct drive of the illness experience and the health anxiety, the illness coping behavior pathway of the urban empty-nest older adults presents as a self-reinforcing cycle from self-medication to seeking medical treatment, and it exhibits significant cyclical characteristics.

#### 4.3.1 Stage one: priority of self-medication

Self-medication represents the initial stage in the behavioral pathway and is the primary coping strategy for the empty-nest older adults under the influence of the triggering mechanism. The core behaviors at this stage involve the preventative strategy of medication stockpiling and the practice of self-medication. In terms of preventive strategies, the empty-nest older adults typically stockpile common medications to manage health risks. This behavior is driven by both social and psychological factors. From a practical standpoint, the insufficient intergenerational support compels the older adults to establish their own emergency support system, as described by the respondent R3: “It’s all meds. When you get to a certain age, you gotta have some pills on hand just in case. Do not wanna be caught off guard.” From a psychological perspective, this behavior reflects underlying anxiety about potential future health crises and aging, as the respondent R14 explained: “These days I’m figuring I should save up, for when I really cannot take care of myself anymore.”

In terms of practical behavior, older adults develop an experiential, cognitively based self-medication model. This model is characterized by behaviors such as adjusting medication dosage and frequency independently, as demonstrated by the respondent R16’s approach to treating a cold: “Do not even bother reading the instructions. If your cold gets really bad, just take more.” It also includes self-prescribing medications, as described by the respondent R4: “I take AXX and OXX, Sometimes I have some of that GXX, And if my blood pressure goes up, I’ll toss in some GXX.” Additionally, some older adults engage in polypharmacy, as the respondent R13 explained: “Besides the OXX, I’m also on GXX for my angina, plus meds for my blood pressure.” For common insomnia issues, interview data revealed a pattern of alternating medications, as described by R10: “I alternate between these three meds so I do not become dependent.” There is also the phenomenon of taking medication without a diagnosis, as the respondent R9 noted: “The accessibility of medication plays a significant role in shaping these behaviors; older adults tend to prioritize medications that are easily available, particularly for insomnia, as R5 pointed out: “ZXX is hard to get, but EXX and AXX can be found easily, so I usually buy EXX and AXX.” The degree of “substitution” of this self-medication for conventional medical treatment is closely related to the subjective perception of pain in the older adults. For mildly tolerable discomfort, it often completely replaces medical treatment; for symptoms that remain troublesome but are still bearable, it serves as a means of partial substitute and a means of disease exploration; when the perception is strong and unbearable, it acts as an emergency buffer before medical treatment. In the end, if the symptoms cannot be contained, it will push its behavior to the next stage.

#### 4.3.2 Stage two: consulting community networks for medication advice—coping adjustment through social networks

When the self-medication proves ineffective, the older adults move to the consulting community networks for medication advice stage, where they seek advice or coping strategies through their social networks (peer groups, community, and online platforms).

##### 4.3.2.1 Peer-group-based medication inquiry—experience sharing and emotional support

Peer groups serve as a core source of support. The older adults share experiences and offer emotional support by discussing their illnesses and medications. For example, the respondent R16 shared a heart issue experience: “A sharp pain in my heart woke me up some night. I texted my fishing buddy, and turns out he has the same condition. His advice was to take some DXX.” The peer groups also provide alternative medical treatment information, such as the respondent R14’s experience: “Actually, trying that moxibustion chair was my sister’s idea. You know, most people aren’t really keen on going to the hospital; they’d rather go for a moxa session.”

##### 4.3.2.2 Community-based medication inquiry—dependence on convenience and limited professionalism

Community clinics and pharmacies, due to their convenience, become important channels for medication, but they inherently exhibit characteristics of “convenience dependence” and “limited professionalism.” Some older adults rely heavily on community clinics to prescribe medications, as the respondent R21 noted: “I mostly get my prescriptions from community clinics.” However, the diagnostic capabilities of community clinics are often limited by insufficient equipment and a shortage of medical specialists. The respondent R1’s experience illustrates this: “Last year, I was getting a drip IV for a few days at the local community clinic. But I ended up getting even worse, so the doctor just told me to go to a major hospital.” Similarly, the convenience of local pharmacies further reinforces medication dependence, as the respondent R7 described: “I’ll just go down to the pharmacy and buy it.”

##### 4.3.2.3 Online medication inquiry—the coexistence of information empowerment and cognitive traps

The internet, as a new channel, has a paradoxical impact. Some older adults obtain medication information or treatment knowledge through short videos or live streaming platforms. For example, the respondent R15’s behavior was: “I saw something about a medication for that on TikTok. I’ll check it out at the drugstore sometime and maybe give it a shot.” While this may improve health management abilities of the older adults, it also carries the risk of inappropriate medication use and the potential adverse health outcomes stemming from misinformation.

The non-professional information on which community-based medicine relies has experience limitations and accuracy risks. When symptoms persist or worsen after taking drugs recommended by the community, the older adults will be prompted to turn to the formal medical system for solutions.

#### 4.3.3 Stage three: seeking medical treatment—the process of ambivalence, characterized by coexisting need and resistance

When the self-medication and the consulting community networks for medication advice inquiries fail to alleviate symptoms, the older adults ultimately enter the seeking medical treatment stage. This stage exhibits strong contradictions—there is a clear need for medical treatment (to obtain effective treatment), but it is accompanied by significant resistance to seeking medical treatment. Seeking medical treatment is primarily driven by multiple inhibiting factors:

##### 4.3.3.1 Economic burden inhibition

The payment structure of “heavy hospitalization and light outpatient” in the public medical insurance system leads to higher out-of-pocket costs for outpatient treatment, which significantly inhibits the willingness to seek medical treatment (41). The interviewee R1’s concerns directly reflect this dilemma: “Medicare hospitalization can be used, and the prescription seems to have to be cash.” It is more realistic that many commonly used drugs can be easily purchased in pharmacies, and their prices are not significantly different from those in hospital pharmacies. In addition, hospital visits also need to pay additional registration fees, consultation fees, and endure long queues. These factors together push the older adults toward self-medication. Interviewee R15 said, “go to a hospital, just registered in line for most of the day, the drug price is not cheaper than the pharmacy, it is better for me to buy some medicine to eat.”

##### 4.3.3.2 Barriers to accessing high-quality healthcare

The uneven distribution of medical resources and the complexity of the medical treatment process significantly increase the difficulty of seeking medical care. The respondent R16’s experience is a representative example: “I went on the first day, and they told me to come back the next day. So, I showed up at 7 a.m., but did not get to see the doctor until 10 a.m.” Additionally, the phenomenon of “relationship-based medical treatment” is widespread, further exacerbating the marginalization of older adults without social capital-based medical privilege. The respondent R18 emphasized the importance of connections: “Every time, I have to ask my buddy’s wife for help. She’s a head nurse. I call in a favor so she can hook me up with the prescription. Otherwise, I just cannot get my meds.”

##### 4.3.3.3 Doctor-patient trust crisis

Some older adults experience “over-testing” or perceive “ineffective treatment,” leading to a significant gap between expected and actual treatment outcomes. A compelling example is the respondent R16’s disappointed experience: “My girl took me to see a specialist at the hospital. We ended up paying a thousand bucks, and they could not even figure out what was wrong.” This gap triggers doubts about the doctor’s professional abilities and, at times, leads to the criticism of broader flaws within the medical system. The erosion of trust often drives older adults to retreat to a perceived ‘safety zone’ constituted by self-medication and seeking medication advice from their community.

#### 4.3.4 Result feedback—the closed loop of health management and the restart of the cycle

Result feedback constitutes the dynamic closed-loop mechanism of the illness coping behavior. The result feedback also serves as the starting point for triggering a new cycle of coping behaviors. This stage both continues the previous behavioral pathway and reflects the reconstruction of health capital. After temporarily alleviating symptoms through self-medication, consulting community networks for medication advice, or seeking medical treatment, empty-nest older adults implement daily health management to consolidate the effects. This includes the regular health monitoring, embedding health maintenance behaviors into their daily routines, and continuously accumulating health knowledge. The effectiveness of health management marks the conclusion of the previous illness coping behavior; new health issues encountered during the management process then serve as the trigger for a new cycle of coping behavior, restarting the loop. The core mechanism lies in the fact that the older adults apply the “successful experiences” gained from the previous coping round to address new problems, creating an iterative loop in their behavior pathway. Specifically, when passive medical treatment is frustrated by economic burden, cumbersome process, or doctor-patient trust crisis, this frustration will strengthen the cognition that self-medication or community-based medication is more reliable and controllable. The emotional comfort and experience recognition obtained by community medicine consolidate the “sense of security” obtained by non-professional medical approaches. The continuous deepening of the older adults’ alienation from the public health system and their dependence on self-coping strategies makes “self-medication” the first choice for subsequent health problems, forming a logical closed loop of self-medication.

## 5 Conclusion

### 5.1 The behavioral closed loop of “substituting self-medication for formal medical treatment”—the core logic of illness coping

This study reveals that the illness coping behavior of urban empty-nest older adults forms a medication-centered behavioral logic closed loop, which is the core characteristic of their health behavioral pattern. Based on their personal observations and experiences, socially circulated disease knowledge, and fragmented biomedical knowledge, older adults develop lay illness explanatory models (42) that guide their primary choice of self-medication. This is particularly evident in the preventive strategy of medication stockpiling and the practice of self-medication. When the self-medication proves ineffective, the older adults often resort to consulting community networks for medication advice, seeking adjustments through peer experience sharing, community resources, and online channels. This marks a shift from individualized coping to social network support. However, this “substituting self-medication for formal medical treatment” pathway contains a significant paradox: although the non-professional self-medication offers immediacy and low cost, it can lead to misdiagnosis, medication dependency, or the escalation of health risks due to cognitive biases and limited information. Consequently, it can adversely aggravate the disease burden, running counter to its intended health purposes. The operational mechanism of this behavioral closed loop is rooted in the joint influence of preconditions and triggering mechanisms, unfolding through the self-reinforcing cycle of “self-medication → consulting community networks for medication advice → Seeking medical treatment,” and ultimately restarting the cycle through feedback from the health management.

### 5.2 Intergenerational transformation and medical structure—socio-cultural drivers of behavioral logic

The deep motivation of urban empty-nesters “taking medicine for treatment” is rooted in the structural contradiction between the transformation of social intergenerational relations and the medical system. From the perspective of social and cultural construction, the weakening of traditional family pension has enabled the older adults to reconstruct “self-reliance” and “drug autonomy” into a dignity narrative that conforms to modernity. This is a cultural expression of the forced transfer of health responsibility to individuals in the “paradox of medical familism.” At the structural level, through the “social ladder” effect of “biological power” operation and resource distribution, the medical system systematically excludes the accessibility of continuous and comprehensive medical services for the older adults at the grass-roots level, forcing them to retreat to convenient and non-professional self-management. In the face of this double pressure, the medication behavior of the older adults can be regarded as a kind of “local biology” practice, that is, they develop local survival strategies to cope with physical decline and medical barriers in the interaction of their own experience, local resources and global medical information. Therefore, “medication” is not only the embodiment of the initiative of the older adults under structural constraints, but also exposes their health vulnerability-it is not a simple irrational choice, but a risky path dependence formed by the individual under the macro social ecological squeeze. The solution must jump out of the medical level and focus on the reconstruction of the social support network and the rational allocation of health resources.

### 5.3 Internalization of self-reliance and peer-group-based mutual support—psychological and collective coping mechanisms of the older adults

The illness coping behavior is fundamentally a psychological defense mechanism and a social adaptation strategy for empty-nest older adults in the face of structural constraints. Psychologically, “substituting self-medication for formal medical treatment” serves as an autonomous practice to resist the erosion of social roles: the older adults reconstruct control over their health through medication stockpiling and self-medication, avoiding being labeled as “dependent,” and thus preserving their dignity as elders. This commitment to “self-reliance” imbues them with the symbolic role of “active copers,” acting as a psychological defense against the anxiety of aging. Socially, peer-group-based mutual support networks fill the void left by intergenerational support: peers offer emotional empathy, experience sharing, and alternative medical information, creating a health management model that operates on both “internalization of self-reliance” and “peer-group-based mutual support” This network highlights the potential of “older adult helping older adult” as a community resource, though its non-professional nature also introduces risks, such as misdiagnosis. This mechanism demonstrates that disease coping is not only a medical behavior but also a process of meaning construction, wherein older adults rebuild social connections and maintain their dignity in the context of modernization.

## 6 Research implications and limitations

### 6.1 Research implications—structural reflection and subjective care in health sociology

Beyond individual attributions, it is crucial to reconstruct the health support system. From a health sociology perspective, health issues are deeply rooted in social structures. The medication dependency behavior of empty-nest older adults is not simply the result of “Limited health literacy” or “Outdated health-seeking beliefs,” but rather the consequence of the combined effects of “insufficient intergenerational support” and “structural contradictions within the healthcare system.” Therefore, policy interventions must go beyond addressing individual behaviors and focus on rebuilding social support networks and optimizing healthcare resource allocation. This may involve strengthening the community’s role to provide a bottom-line guarantee of emotional support and health management. and the basic healthcare services. For instance, establishing a community pharmacist medication consultation and review system and a regular pairing assistance mechanism with social workers. Shift medical insurance policies to prioritize the outpatient care, the chronic disease management, and the primary healthcare, which can effectively reduce economic burdens and explore innovative models such as “time banks” or “peer-group-based mutual support” to fill the gap in intergenerational support and build more resilient community health systems.

Empowering, rather than substituting: Respecting older adults autonomy and local knowledge. The study reveals that “substituting self-medication for formal medical treatment” serves as a psychological defense and social adaptation strategy for older adults under structural constraints. Health interventions should respect their autonomy, avoiding the treatment of the older adults as passive recipients. The Policy planning should focus on “empowerment.” On the other hand, it is essential to acknowledge both the value and limitations of “lay health knowledge” formed through peer networks. Rather than completely dismissing it, scientific guidance should be provided through community health education. For example, implementing peer-education-based health promotion programs for safe medication use among the older adults can transform informal support networks into positive forces for health promotion.

Shifting from Disease Treatment to Building a Health-Oriented Doctor–Patient Relationship. The study highlights that the doctor-patient trust crisis is a key barrier to medical treatments. This suggests that strategies for healthy aging should shift from simply treating diseases to systematically constructing the Health-Oriented Doctor–Patient relationships. Within the medical system, there is an urgent need to strengthen the capabilities of primary care doctors and improve service awareness, optimizing the medical treatment process and rebuilding doctor-patient trust. At the societal level, it is essential to promote active intergenerational interactions, alleviate the older adults’ “burden perception,” and encourage families, communities, and healthcare institutions to collaborate in forming supportive networks. This would help create a social environment conducive to older adults seeking professional help.

### 6.2 Research limitations

This study has certain limitations. First, as a grounded theory study based on interviews with 25 empty-nest older adult individuals in District L of City H in China, while the sample size is adequate, all participants are urban residents in China, presenting geographical and cultural constraints. Future research will expand to rural areas in China, groups with different socioeconomic backgrounds, and cross-cultural comparisons to enhance the generalizability of the findings. For example, examining its applicability in East Asian societies such as Japan and South Korea, which share a Confucian filial piety culture but differ in family policies and welfare systems. Second, although this study touches upon the phenomenon of “online medication inquiry,” it does not deeply explore the impact of the digital divide on certain empty-nest older adults. Subsequent research will investigate how the proliferation of smart technologies (e.g., artificial intelligence [AI] diagnosis and wearable devices) might potentially reshape this “cycle.” Third, constrained by the bottom-up nature of qualitative methods, this study provides a relatively limited in-depth exploration of the broader medical marketization mechanisms. The shaping effects of macro-level institutional factors on “using medicine as medical treatment” behaviors require further analysis through mixed methods in future research.

## Data Availability

The original contributions presented in the study are included in the article/supplementary material, further inquiries can be directed to the corresponding author.

## Footnotes

1 Healthy aging is an important national strategy of China and a common task, requirement, and responsibility of all Chinese people. Its importance extends beyond the wellbeing of hundreds of millions of older adult people in China; it also profoundly affects the harmony and stability of Chinese society and the high-quality development of the economy.

## References

[1] Zhao J Liu Z. The impact of internet use on the health of the elderly. Chin J Popul Sci. (2020) 5:14–26+126. Available online at: https://kns.cnki.net/kcms2/article/abstract?v=HHZEz91P5iLY4XzEeJ4806kWQtzxrDjlrYNH1Ghexb9SoKWUCce0iTnMwdt0oCzUWISOnsKj0l8TZGH6yy81ThJ97Wk8J9hRo29NWCG_pthg6X-2y_UwFQgFNLHOxFMOQ-I1yAD2P8X6XkCAFMPkb36ZpC08WFWLFnORXV18YJHaIaSyKKoRMA==&uniplatform=NZKPT&language=CHS

[2] Li J Li J. Life quality of urban empty-nested elderly. Popul J. (2012) 3:31–41. doi: 10.3969/j.issn.1004-129X.2012.03.004

[3] Li M Hao S. Research progress on the health status of empty nesters in China. Chin J Geriatr Care. (2023) 21:103–6. doi: 10.3969/j.issn.1672-2671.2023.05.022

[4] He S Wen H Hou J Wen K Zhang Y. Current status and influencing factors of life quality of empty-nest elderly in Harbin communities. Med Soc. (2024) 37:30–36+79. doi: 10.13723/j.yxysh.2024.06.005

[5] Huang WJ Fu GS Tan LN Li CZ Wei QL Gao Q . Self-esteem, life satisfaction and their influencing factors among urban empty-nest elderly. Chin J Gerontol. (2021) 41:1326–9. doi: 10.3969/j.issn.1005-9202.2021.06.056

[6] Glick PC. The family cycle. Am Sociol Rev. (1947) 12:164–74. doi: 10.2307/2086982

[7] Din Y Yan C Ma X Liu X Pan F. Analysis of anxiety and depression status and influencing factors among community empty-nest elderly. Anhui Med J. (2019) 40:947–50. doi: 10.3969/j.issn.1000-0399.2019.08.030

[8] Li N Zhao C Gao L. Analysis of the prevalence of chronic diseases and its influencing factors among rural left-behind elderly. Med J Chin People's Health. (2015) 27:76–8. doi: 10.3969/j.issn.1672-0369.2015.03.041

[9] Qi Y Xiong Y. Study on dynamic bidirectional relationship between physical and mental health among the middle-aged and elderly people in China. Med Soc. (2024) 37:05–111. doi: 10.13723/j.yxysh.2024.01.016,

[10] Li Y Wu M. Cognitive frame of self-treatment. Chin Health Econ. (2011) 30:18–21. doi: 10.3969/j.issn.1003-0743.2011.01.007

[11] Wen X Lv W. The effect of biased health perception on self-health care utilization among middle aged and elderly: evidence from China. China Econ Stud. (2024) 5:164–79. doi: 10.19365/j.issn1000-4181.2024.09.11

[12] Yan X Chang F Lu Y Yang Y. Influence of self-medication on medical expenditure and health status of the elderly in China: empirical analysis based on2018CHARLS data. China Pharm. (2022) 33:2438–42. doi: 10.6039/j.issn.1001-0408.2022.20.02

[13] Zhang F Guo G. Investigation and analysis of elderly people’s medication knowledge. Shandong Med. (2003) 2:10–2. doi: 10.3969/j.issn.1002-266X.2003.02.005

[14] Bennadi D. Self-medication: a current challenge. J Basic Clin Pharm. (2014) 5:19–23. doi: 10.4103/0976-0105.128253, 24808684 PMC4012703

[15] Gong K Yu Z Ma Y. Self-management of health care for elderly people. Chin J Gerontol. (2012) 32:4591–4. doi: 10.3969/j.issn.1005-9202.2012.20.126

[16] Han Z Xu A. CiteSpace-based visualization analysis of geriatric digital health management research. Chin Health Serv Manag. (2024) 41:1194–200. Available online at: https://kns.cnki.net/kcms2/article/abstract?v=HHZEz91P5iLGK6XyNvdYLzj87gJGq8TIck9votg2KTt_3XtfZ14QU5OQv5hAYFfRSpgYw7FZD1D08BQEUtbDozysCyG8WJU7jmiy6_hynIWp44tINiBLY9eRYVt4JnksVGyD_QFc7L4CarCBbaiv5p68Xbpk6sIdnUuugfqLNA-sNfTgEfsNeA==&uniplatform=NZKPT&language=CHS

[17] Shi Z. Research on mechanism of influence of internet use situation on proactive health consciousness among middle-aged and elderly people in China. Med Soc. (2024) 37:37–44. doi: 10.13723/j.yxysh.2024.06.006

[18] Yin P Huang Y Wu K. The dilemmas and countermeasures of smart elderly care services from the perspective of digital social work. Jinyang Acad J. (2025) 5:30–44. doi: 10.16392/j.cnki.14-1057/c.2025.05.005

[19] Zou H Jiang D Zhang L. Recent advances in evaluation tools and associated factors for patient delay in chronic disease patients. Chin Gen Pract. (2022) 25:893–8. doi: 10.12114/j.issn.1007-9572.2021.00.296

[20] Wang X Guan X Zhang D. Patient delay and associated factors in older adults with multimorbidity. Chin Gen Pract. (2024) 27:2505–11. doi: 10.12114/j.issn.1007-9572.2023.0614

[21] Guo B Cheng H Zhang W Liu Y Wang C Liu C . Utilization and influencing factors of health service of urban elderly in Heilongjiang Province. Med Soc. (2019) 32:1–4. doi: 10.13723/j.yxysh.2019.06.001

[22] Ren Q Ren X. Effect of intergenerational support from children on older adults’ healthcare seeking behaviors. Sichuan Univ J (Med Edn). (2023) 54:614–9. doi: 10.12182/20230560505, 37248593 PMC10475417

[23] Li C Wang P. Study on the impact of medical service accessibility on the treatment of chronic diseases in the elderly. Chin Health Serv Manag. (2020) 37:592–595, 610. Available online at: https://kns.cnki.net/kcms2/article/abstract?v=HHZEz91P5iJxi9ZT8FOy-dp-TJBZ2YIa9Bfz_qE97fJ8uyKTsvBXoOySfHaufSO8rtaDLKD8MMJ4_MPFlRCLLr2-03YXbsCMgrz4JErPF2PWzaIrfjltUwrJ1VjfyXeFpZ3CGV16N5zabYTrglQdRM-fCNo_eqOQcECUhNkKtAkdk17gz3kwkg==&uniplatform=NZKPT&language=CHS

[24] Jin S Chen J Wang C. Aging support dilemmas and interventions for China’s rural empty-nest seniors. Chin J Gerontol. (2017) 3:749–50. doi: 10.3969/j.issn.1005-9202.2017.03.100

[25] Jing J. Medical social ecology viewed from death narrative. Thinking. (2022) 48:105–17. doi: 10.3969/j.issn.1001-778X.2022.01.011

[26] Rosenstock IM Strecher VJ Becker MH. Social learning theory and the health belief model. Health Educ Q. (1988) 15:175Y183. doi: 10.1177/109019818801500203 3378902

[27] Andersen RM. Revisiting the behavioral model andaccess to medical care:does it matter? J Health Soc Behav. (1995) 36:1–10. doi: 10.2307/2137284 7738325

[28] Strauss A Corbin J. Basics of qualitative research: grounded theory procedures and techniques Translated by Zonggu Xu. Taipei: Giant Flow Publishing (1998).

[29] Strauss A Corbin JM. Basics of qualitative research: Grounded theory procedures and techniques. Newbury Park: Sage Publications (1990).

[30] Gong HX Li HY. Summary and prospects of research on family intergenerational support in China. Educ Observ. (2022) 11:42–6. doi: 10.16070/j.cnki.cn45-1388/g4s.2022.36.01

[31] Sun BL Ren XH. Research on the impact of intergenerational support from children on older adults' behaviors of seeking medical care under the perspective of urban-rural disparities. Health Soft Sci. (2025) 39:90–5. Available online at: https://kns.cnki.net/kcms2/article/abstract?v=HHZEz91P5iI4-hWUAPrbL4KdM9YdPKvxDpELkzXX-hB4P4slTPSEHZDEBo4CoRH5vtA1n9s_iGLrPc-f6Hd4ooDjhRcquNMBsigSiVSMpgpr_3z10u1cdRWcZ9Z22fQcHARaw_olBC37wkdJfQ84oOFO6OceXrqd-k2yDwlGZ2UV1Kjd41QXkQ==&uniplatform=NZKPT&language=CHS

[32] Huang Q Du P Chen G. The intergenerational support between adult children and older adults and its associated factors. Popul Dev. (2018) 24:10. Available online at: https://kns.cnki.net/kcms2/article/abstract?v=HHZEz91P5iJw9S1tK9dpJSsm50s4nSQGqFGLdrVdZDnfo6y8Z6cLAhjczXRxPbBS-87gB345a6c2ZLzCH3SJzN07wj9uneulnCyMwD6RySoMQ5PwhkB1gmH0lC-hjqb3NqaNLA1seo3JD9zFYWCZk5IRG9dWTB8HHjGmq1dpjrpebwhqQ_FZEg==&uniplatform=NZKPT&language=CHS

[33] Hu Y Zang H Yang B. The impact of long-term care insurance on inter-generational support in rural areas. South China Popul. (2025) 40:34–45. doi: 10.3969/j.issn.1004-1613.2025.01.004

[34] McPherson CU Wilson KG Murray MA. Feeling like a burden: exploring the perspectives of patients at the end of life. Soc Sci Med. (2007) 64:417–27. doi: 10.1016/j.socscimed.2006.09.013 17069943

[35] Cousineau N McDowell I Hotz S Hebert P. Measuring chronic patients' feelings of being a burden to their caregivers: development and preliminary validation of a scale. Med Care. (2003) 41:110–8. doi: 10.1097/00005650-200301000-00013, 12544548

[36] Kleinman A. The illness narratives: Suffering, healing, and the human condition. New York: Basic Books (1988). 10.1097/ACM.0000000000001864 28952997

[37] Ferrell R Dean G. The meaning of cancer pain. Semin Oncol Nurs. (1995) 11:17–22. doi: 10.1016/S0749-2081(95)80038-7, 7740218

[38] Yu C Li W Deng M. Hope and anxiety: the study of female embodied experience with ARTs. Chin J Sociol. (2019) 39:84–115. doi: 10.15992/j.cnki.31-1123/c.2019.04.004

[39] Tu J Zhong J. The body, self and identity of esophageal cancer patients. J Guangxi Univ Natl (Philos Soc Sci Edn). (2017) 39:36–45. Available online at: https://kns.cnki.net/kcms2/article/abstract?v=HHZEz91P5iIYe5y6P0yh9AOsvYYY4RiL2dwJq7Y921Vu8bzPCtbRdf4okZRqm6rBTTI-qzyzZc2l0lUh4pFPmoyEacqJe2h74iv6ub8X4eU0EKfeo5NruHop0jNKbMhotfKPfaYuaLAhI23vCvt1cBoypaYYBhaAF62QgupLOH-zAmOfiOMCDQ==&uniplatform=NZKPT&language=CHS

[40] Tu J Cheng Y. Disease interpretation of esophageal cancer patients: understanding, legitimization, and meaning-seeking. Thinking. (2016) 42:52–60. doi: 10.3969/j.issn.1001-778X.2016.03.008

[41] Li SS Chen ZY. Study on the influence of medical insurance on the healthcare seeking behavior of the elderly based on the com parative analysis of outpatient and inpatient behaviors. Chin Health Resources Manag. (2022) 39:823–8. Available online at: https://kns.cnki.net/kcms2/article/abstract?v=HHZEz91P5iKghzH-Bnwl_soWEs-tQk6hyYjB6KmO6TN_H3RUkcls7lpcSIbk_3i-_iCAbXo6lwUXfFMqKDY1rGKhxQ-MgMIXxbAu04dVhjugQUpOgEd_j7ZNu0G7dUvPSkya2GW-3T_P3TyGCFTslW2yfil1ZOnyv0n_DHCbPtJ2T2brve9fmg==&uniplatform=NZKPT&language=CHS

[42] Kleinman A. Patients and healers in the context of culture: an exploration of the borderland between anthropology, medicine, and psychiatry Berkeley: University of California Press (1980).

